# Association of Elevated Maternal Serum Total Bile Acids With Low Birth Weight and Intrauterine Fetal Growth Restriction

**DOI:** 10.1001/jamanetworkopen.2021.17409

**Published:** 2021-07-19

**Authors:** Fuzhen Song, Yuanyuan Chen, Lei Chen, Huan Li, Xiajin Cheng, Weibin Wu

**Affiliations:** 1 The International Peace Maternity and Child Health Hospital, Shanghai Jiao Tong University School of Medicine, Shanghai, China; 2 Shanghai Key Laboratory of Embryo Original Diseases, Shanghai, China; 3 Hongqiao Street Community Health Service Center, Changning District, Shanghai, China; 4 Department of Gynecology and Obstetrics, Songjiang Maternity and Child Health Hospital, Shanghai, China

## Abstract

**Importance:**

Bile acids play essential roles in metabolic modulation. Excessive serum total bile acid (sTBA) levels during pregnancy are associated with adverse perinatal outcomes; however, their association with the risk of intrauterine growth restriction (IUGR) remains unclear.

**Objective:**

To investigate the association between maternal sTBA concentration during pregnancy and the risk of IUGR.

**Design, Setting, and Participants:**

This retrospective cohort study included pregnant individuals who delivered live singleton neonates and had regular antenatal examination records available at a hospital-based center in Shanghai, China, from 2014 to 2018. Data were analyzed from July to November 2020.

**Exposures:**

Maternal sTBA concentration during pregnancy.

**Main Outcomes and Measures:**

Fetal birth weight and probability of low birth weight (LBW) and IUGR.

**Results:**

This study included 68 245 singleton pregnancies with live births for analysis. The mean (SD) age of the pregnant individuals was 30.5 (3.8) years, 67 168 patients (98.4%) were Han, and 50 155 (73.5%) were nulliparous. Nonlinear regression models suggested that there was an inverted J-shaped association between maternal sTBA level during pregnancy and fetal birth weight, with a steep decrease in birth weight at high sTBA levels (estimated mean [SE] birth weight for sTBA of 40.8 ug/mL, 2879 [39.9] g) and greater birth weights at lower sTBA levels (estimated mean [SE] birth weight for sTBA 0.4 μg/mL, 3290 [3.9] g; and for 4.1 μg/mL, 3334 [1.6] g). Lower birth weight and a higher incidence of IUGR were observed in patients with gestational hypercholanemia (sTBA ≥4.08 μg/mL) compared with those without gestational hypercholanemia (birth weight: estimated adjusted mean [SE], 3309 [3.32] vs 3338 [0.80] g; *P* = .005; incidence of IUGR: 62 of 4467 [1.4%] vs 312 of 63 778 [0.5%]; *P* < .001). Moreover, compared with patients with sTBA concentrations of less than 4.08 μg/mL, those with gestational hypercholanemia had an increased risk of LBW (adjusted odds ratio [aOR], 1.29; 95% CI, 1.09-1.53) and IUGR (aOR, 2.18; 95% CI, 1.62-2.91). In addition, there was an additive interaction between hypertensive disorders in pregnancy (HDP) and hypercholanemia on LBW and IUGR risk. The highest risks of LBW and IUGR were found in pregnant individuals with both HDP and hypercholanemia compared with those with normotensive pregnancies with sTBA concentrations less than 4.08 μg/mL (LBW: aOR, 9.13; 95% CI, 6.88-12.12; IUGR: aOR, 19.14; 95% CI, 12.09-30.28).

**Conclusions and Relevance:**

This study found that gestational hypercholanemia was associated with an increased risk of LBW and IUGR, especially in pregnant individuals with HDP. Therefore, it would be meaningful to monitor sTBA concentration during the follow-up of pregnancies with potential IUGR.

## Key Points

**Question:**

Are elevated maternal total bile acid concentrations associated with low birth weight and risk of intrauterine growth restriction (IUGR)?

**Findings:**

In this retrospective cohort study that included 68 245 singleton pregnancies, elevated maternal serum total bile acid concentration was associated with an increased risk of low birth weight and IUGR, and this risk appeared to be higher when coexisting with hypertensive disorders in pregnancy.

**Meaning:**

These findings suggest that clinicians should monitor bile acid concentration during the follow-up for pregnancies with potential IUGR.

## Teaser

This cohort study investigates the association between maternal serum total bile acid concentrations during pregnancy and the risk of intrauterine growth restriction.

## Introduction

Bile acids play essential roles in intestinal lipid absorption and hepatic metabolic modulation.^1^ Dysregulated bile acid levels in pregnancy can lead to the development of intrahepatic cholestasis of pregnancy (ICP), which is a pregnancy-specific complication characterized by elevated serum total bile acid (sTBA) concentrations (also known as hypercholanemia), pruritus, and abnormal liver transaminase levels. It affects between 0.2% and 5% of pregnant individuals worldwide, with regional differences.^2,3^ ICP is relatively benign for mothers, whose symptoms and abnormal laboratory results recover spontaneously after delivery.^1^ However, it can cause severe adverse outcomes for fetuses, including preterm labor, fetal hypoxia, stillbirth, and perinatal death,^4,5^ and predispose them to metabolic diseases in later adult life.^6^ Although the definition of ICP and gestational hypercholanemia is still debated, it is well-accepted that the severity of adverse outcomes of ICP is closely associated with the maternal sTBA level. As reported, the incidence of fetal asphyxia, preterm labor, and meconium-stained amniotic fluid was much higher in pregnant individuals with sTBA concentrations of 16.3 μg/mL or higher,^4^ whereas the risk of stillbirth was markedly increased in patients with sTBA concentrations of 40.8 μg/mL or higher (to convert sTBA to micromoles per liter, multiply by 2.448).^5^

Intrauterine growth restriction (IUGR) is a pathological condition in which a fetus is unable to achieve its genetic growth potential for pathologic reasons during intrauterine development.^7,8^ IUGR is a nonnegligible complication that can also cause serious adverse perinatal consequences, including stillbirth, preterm birth, and neonatal morbidity.^9,10^ Moreover, IUGR is associated with an increased risk of fetal-originated metabolic syndrome and cardiovascular disease.^10,11,12^ Previous studies^7,13,14^ have reported that placental insufficiency, intrauterine infection, fetal chromosome anomalies, gestational hypertension, and preeclampsia are risk factors for IUGR. However, the etiopathology of IUGR has not been fully elucidated.

Several observational studies^15,16^ have reported an association of excessive sTBA with lower birth weight as well as an increased risk of small for gestational age (SGA). However, it has also been reported that an increased incidence of large for gestational age (LGA) neonates was found in pregnancies complicated by ICP.^17,18^ Additionally, these reports were mostly based on European or US populations, while very few data on Asian populations were available. Thus, the association of sTBA solely or with other gestational complications with fetal weight remains largely undefined. Moreover, there is no epidemiological evidence to identify an association between sTBA level and IUGR in human pregnancies. We conducted a large retrospective cohort study to investigate the association between maternal sTBA concentration and the risk of IUGR.

## Methods

### Study Design and Population

A retrospective cohort study was conducted in a university-affiliated teaching hospital, the International Peace Maternity and Child Health Hospital (IPMCH), Shanghai Jiao Tong University School of Medicine in Shanghai, China. Data were collected from January 2014 until December 2018 through the hospital electronic medical record system. This study was approved by the Institute Medical Ethics Committee of IPMCH with exemption from informed consent due to the retrospective nature of the study. The protocol was registered at the Chinese Clinical Trial Registry (ChiCTR2000034742). This study followed the Strengthening the Reporting of Observational Studies in Epidemiology (STROBE) reporting guidelines for retrospective cohort studies.

Eligible mother and neonate pairs were those who underwent first-trimester prenatal screenings and delivered live births at IPMCH. A total of 76 129 patients with regular antenatal examination records were initially included in this study. Subsequently, the following were excluded from further analysis: (1) pregnant individuals with severe hepatic dysfunction (ie, alanine transaminase [ALT] or aspartate aminotransferase level >100 U/L [to convert to microkatals per liter, multiply by 0.0167]); (2) twin pregnancies; (3) pregnant individuals who underwent in vitro fertilization; and (4) pregnant individuals with history of severe hepatobiliary diseases, including drug-induced liver injury, hepatectomy, fatty liver disease, and gallbladder disease.

### Data Collection, Measurements, and Outcomes

Demographic and obstetrical characteristics, including maternal age, education level, race, parity, prepregnancy body mass index (BMI; calculated as weight in kilograms divided by height in meters squared), history of chronic disease, pregnancy complications, and fetal birth weight were extracted from the medical record system. These data were recorded by gynecologists and nurses through interviews during routine antenatal examinations. Prepregnancy body weight and height were self-reported by participants to calculate prepregnancy BMI.

Data on hepatic biochemistry tests, including sTBA and ALT peak concentrations, were obtained in early pregnancy (8-14 weeks), late pregnancy (28-41 weeks), and peak level during whole pregnancy from the hospital’s laboratory test database. sTBA and ALT concentrations were determined using the enzymatic colorimetric method on a Cobas c702 chemistry analyzer (Roche Diagnostics) according to the manufacturer’s instructions. The intra-assay and interassay coefficients of variation for sTBA and ALT measurements were less than 5%.

Coding of standardized diagnoses was conducted by gynecologists according to *International Statistical Classification of Diseases and Related Health Problems, Tenth Revision*. Data on pregnancy complications, such as diabetes (including preexisting diabetes and gestational diabetes) and hypertensive disorders (including preexisting hypertension, gestational hypertension, and preeclampsia), were extracted from participants’ medical records at discharge after delivery. Preeclampsia and gestational hypertension were defined as having systolic blood pressure of at least 140 mm Hg or diastolic blood pressure of at least 90 mm Hg on 2 occasions combined with or without proteinuria after 20 weeks of pregnancy. Gestational hypercholanemia (or ICP) was diagnosed in pregnancies with sTBA concentration of at least 4.08 μg/mL during pregnancy according to guideline for ICP management of the Chinese Medical Association of Obstetrics and Gynecology.^19^

The primary outcomes were fetal birth weight and IUGR. IUGR was diagnosed following the guideline for IUGR,^20,21^ as indicated by ultrasound-estimated fetal weight or circumference of abdomen smaller than 10% of the birth population after adjusting fetal sex and gestational age and evaluation of potential pathological conditions. Low birth weight (LBW) and macrosomia were defined as fetal birth weight less than 2500 g and birth weight of 4000 g and greater, respectively. SGA and LGA were defined as birth weight of live-born infants below the 10th percentile and greater than the 90th percentile, respectively, adjusting for sex and gestational age according to the Chinese neonatal birth weight reference curve.^22^

### Statistical Analysis

We used a generalized additive model with restricted cubic spline using 3 knots to study the association of sTBA concentrations with birth weight. Furthermore, multiple logistic regression models with restricted cubic spline using 3 knots were used to study the association of sTBA with LBW and IUGR. To improve model assumptions, sTBA concentrations were logarithm transformed in analysis. Further model assumptions were assessed by plotting model residuals and evaluating squared residuals. Analyses were adjusted for potential confounding factors, such as gestational age, maternal age, prepregnancy BMI, race, parity, education level, and fetal sex, based on biological and clinical significance. A variable was identified as a confounder according to reports in previous studies, reduction in the residual variability of the outcome, change of the effect estimates of interest, and other factors. A multiple imputation method (Markov chain Monte Carlo algorithm) was used to process confounders with missing data through creating and pooling 5 imputed data sets, and the descriptive characteristics of imputed and original data sets were not significantly different. All statistical analyses were performed using R version 3.6.3, with packages mgcv, rms, mice, visreg, table 1, and interaction (R Project for Statistical Computing). *P* < .05 was considered statistically significant, and all tests were 2-sided.

## Results

### Clinical Characteristics of the Participants

After exclusions, a total of 68 245 pregnant individuals were included in this study for subsequent analysis (eFigure 1 in the Supplement). The basic characteristics of the study population are shown in Table 1. The mean (SD) age and prepregnancy BMI of the participants were 30.5 (3.8) years and 21.0 (2.7), respectively. In addition, 67 168 participants (98.4%) were of Han race, and 50 155 pregnancies (73.5%) were nulliparous. Missing values included 2224 cases (3.3%) of prepregnancy BMI and 562 (0.8%) of education level. Data were missing at random. The prevalence rates of gestational hypercholanemia, HDP, and diabetes during pregnancy were approximately 6.5% (4467 individuals), 4.3% (2988 individuals), and 12.2% (8328 individuals), respectively. The mean (SD) fetal birth weight was 3340 (429) g. The proportions of LBW, macrosomia, SGA, LGA, and IUGR in this cohort were 2.6% (1779 infants), 5.6% (3851 infants), 4.1% (2778 infants), 10.0% (6817 infants), and 0.5% (374 infants), respectively.

**Table 1.** Basic Characteristics of Study Population

Characteristic	Participants, No. (%)
Maternal characteristics	
Age, mean (SD), y	30.5 (3.79)
Prepregnancy BMI, mean (SD)	21.0 (2.73)
Race	
Han	67 168 (98.4)
Other	1077 (1.6)
Gravidity	
1	35 217 (51.6)
≥2	33 028 (48.4)
Parity	
1	50 155 (73.5)
≥2	18 090 (26.5)
Education level, mean (SD), y	16.1 (2.03)
Delivery method	
Vaginal	39 332 (57.6)
Cesarean	28 913 (42.4)
Gestational hypercholanemia	4467 (6.5)
Hypertension	
Pregnancy induced	2988 (4.3)
Preexisting	882 (1.3)
Diabetes	
Pregnancy induced	8328 (12.2)
Preexisting	32 (0.0)
sTBA concentrations, median (IQR), μg/mL	
Early pregnancy	0.78 (0.53-1.14)
Late pregnancy	1.80 (1.31-2.53)
Peak during whole pregnancy	1.84 (1.39-2.57)
Gestational week for sTBA peak during whole pregnancy, median (IQR), wk	37 (32-39)
Fetal characteristics	
Male sex	35 208 (51.6)
Female sex	33 037 (48.4)
Birth weight, mean (SD), g	3340 (429)
Body length, mean (SD), cm	49.8 (1.28)
LBW	1779 (2.6)
Macrosomia	3851 (5.6)
SGA	2778 (4.1)
LGA	6817 (10.0)
IUGR	374 (0.5)

Abbreviations: BMI, body mass index (calculated as weight in kilograms divided by height in meters squared); IQR, interquartile range; IUGR, intrauterine growth restriction; LBW, low birth weight; LGA, large for gestational age; SGA, small for gestational age; sTBA, serum total bile acids.

SI conversion factor: To convert sTBA to micromoles per liter, multiply by 2.448.

### sTBA Level and Fetal Birth Weight

**Figure 1.**
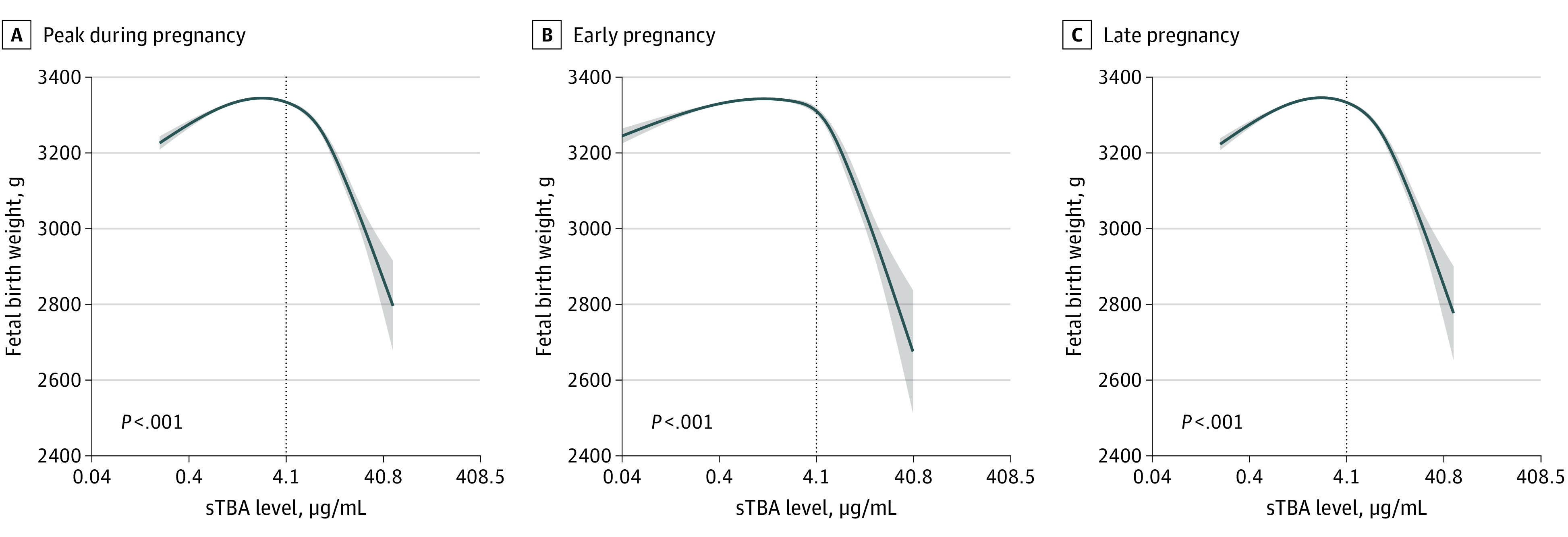
Association Between Maternal Serum Total Bile Acid (sTBA) Levels and Fetal Birth Weight Graphs show the association between peak sTBA level and fetal birth weight during whole pregnancy (A), in early pregnancy (B), and in late pregnancy (C). Data were expressed as estimated mean birth weight with 95% CIs (shaded areas), adjusted for prepregnancy body mass index, age, education level, race, parity, hypertensive disorders in pregnancy, diabetes during pregnancy, fetal sex, gestational age at birth, and alanine aminotransferase level. To convert sTBA to micromoles per liter, multiply by 2.448.

By using a nonlinear regression model, we found that associations between sTBA concentration (peak level during whole pregnancy) and fetal birth weight were an inverted J-shaped. A steep decrease in birth weight was observed at high sTBA levels, with greater birth weights at lower sTBA levels (estimated mean [SE] birth weight: sTBA 0.4 μg/mL, 3290 [3.9] g; 4.1 μg/mL, 3334 [1.6] g; 40.8 μg/mL, 2879 [39.9] g; *P* < .001) (Figure 1A). Moreover, the sTBA level was negatively associated with birth weight in the subpopulation with hypercholanemia (ie, sTBA ≥4.08 μg/mL) using a linear regression model (eFigure 2A in the Supplement). Similar associations were observed between birth weight and sTBA levels in the early and late pregnancy periods (Figure 1B and 1C; eFigure 2B and 2C in the Supplement). The estimated adjusted mean (SE) birth weights comparing patients with sTBA concentrations less than 4.08 μg/mL and 4.08 μg/mL or greater were 3338 (0.80) g and 3309 (3.32) g overall, 3337 (0.81) g and 3318 (3.28) g for measurements from early pregnancy, and 3338 (0.80) g and 3308 (3.32) g for measurements from late pregnancy. Per-unit increases in sTBA levels in whole pregnancy, early pregnancy, or late pregnancy of hypercholanemia subpopulation were associated with a mean (SD) 3.39 (0.71) g, 5.74 (2.00) g, or 3.32 (0.76) g decrease in birth weight, respectively.

Furthermore, fetal birth weight and body length were both lower in pregnant individuals with hypercholanemia than in those without (eTable 1 in the Supplement). The proportions of infants with LBW (164 of 4467 [3.7%] vs 1615 of 63 778 [2.5%]; *P* < .001), SGA (246 [5.5%] vs 2532 [4.0%]; *P* = .004), and IUGR (62 [1.4%] vs 312 [0.5%]; *P* < .001) were higher in individuals with hypercholanemia than without. However, there was no significant difference in the ratio of LGA and macrosomia between these groups. Furthermore, hypercholanemia was associated with increased risk of LBW, SGA, and IUGR (eTable 2 in the Supplement).

### Association Between sTBA Levels and the Risk of LBW and IUGR

Subsequently, we used logistic regression analysis to investigate whether different categories of maternal sTBA levels during pregnancy were associated with the risk of LBW and IUGR. As shown, a higher sTBA concentration (peak level during pregnancy) was associated with an increased risk of LBW and IUGR. Using sTBA level of less than 1.6 μg/mL as a reference, participants with sTBA levels between 8.2 μg/mL and less than 16.3 μg/mL (OR 1.85; 95% CI, 1.24-2.77) and 16.3 μg/mL or greater (OR, 3.23; 95% CI, 1.59-6.56) had higher risks of LBW; participants with sTBA levels of 4.08 μg/mL to less than 8.2 μg/mL (OR, 2.15; 95% CI, 1.52-3.04), 8.2 μg/mL to less than 16.3 μg/mL (OR, 3.91; 95% CI, 2.07-7.37) and 16.3 μg/mL or greater (OR, 5.46; 95% CI, 1.64-18.21) had higher risks of IUGR (Table 2). Similarly, elevated sTBA levels in both early and late pregnancies were associated with increased risks of LBW and IUGR (eTable 3 in the Supplement).

**Table 2.** Analysis of LBW and IUGR Risk in Pregnant Individuals With Different TBA Peak Levels During Pregnancy

sTBA level, μg/mL	No.	LBW		IUGR	
		No. (%)^a^	aOR (95% CI)^b^	No. (%)^c^	aOR (95% CI)^b^
<1.6	39 111	1015 (2.60)	1 [Reference]	165 (0.42)	1 [Reference]
1.6 to <4.1	24 667	600 (2.43)	0.9 (0.81-1)	147 (0.60)	1.28 (1.02-1.61)
4.1 to <8.2	3890	127 (3.26)	1.11 (0.91-1.35)	47 (1.21)	2.15 (1.52-3.04)
8.2 to <16.3	478	28 (5.86)	1.85 (1.24-2.77)	12 (2.51)	3.91 (2.07-7.37)
≥16.3	99	9 (9.09)	3.23 (1.59-6.56)	3 (3.03)	5.46 (1.64-18.21)

Abbreviations: aOR, adjusted odds ratio; IUGR, intrauterine growth restriction; LBW, low birth weight; sTBA serum total bile acids.

SI conversion factor: To convert sTBA to micromoles per liter, multiply by 2.448.

^a^ *P* for trend = .003.

^b^ Adjusted for maternal prepregnancy body mass index, age, education level, race, parity, alanine aminotransferase level, hypertensive disorders of pregnancy, and diabetes during pregnancy.

^c^ *P* for trend < .001.

### Additive Association of Hypercholanemia and HDP With the Risk of LBW and IUGR

**Figure 2.**
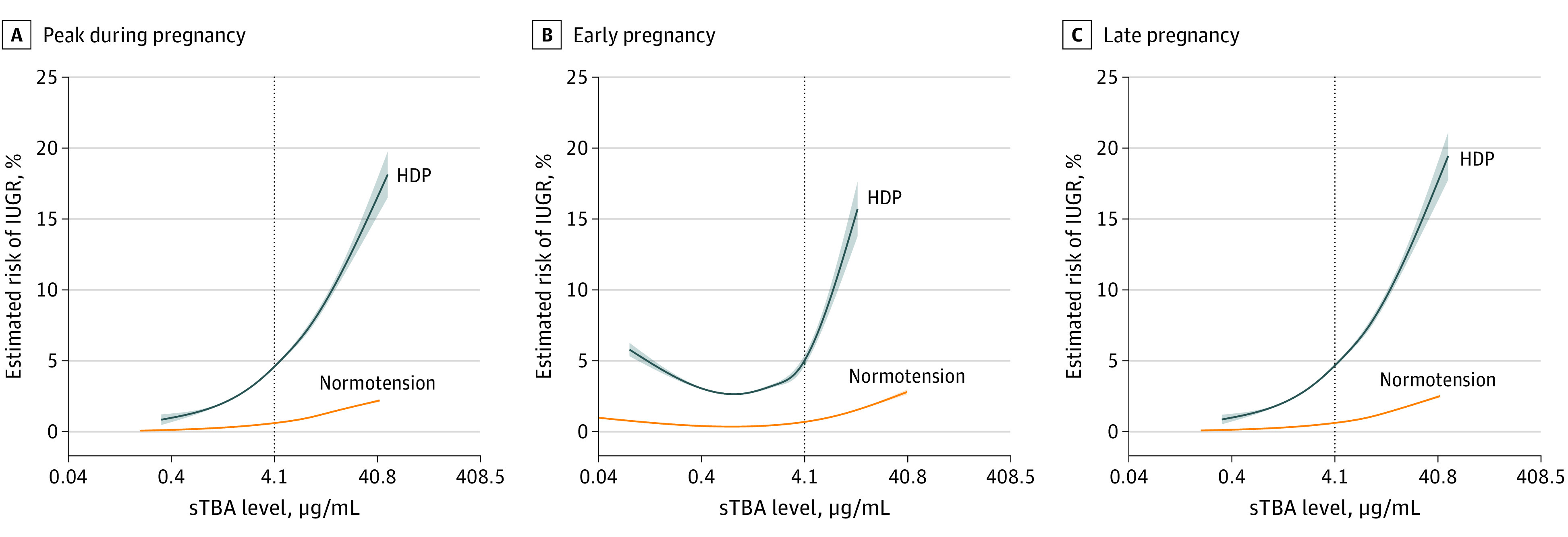
Association Between Maternal Serum Total Bile Acid (sTBA) Levels With Risk of Intrauterine Growth Restriction (IUGR) Stratified by Hypertensive Disorders in Pregnancy (HDP) Association of risk of IUGR with peak sTBA level during whole (A), early (B), and late (C) pregnancy periods was stratified by HDP conditions. Data are expressed as estimated mean risk with 95% CIs (shaded areas) and adjusted for prepregnancy BMI, age, education level, race, parity, HDP, diabetes during pregnancy, and alanine aminotransferase level.

Subsequently, the association of sTBA and risks of LBW and IUGR was stratified by prepregnancy BMI (eTable 4 in the Supplement) and HDP status (eTable 5 in the Supplement). Hypercholanemia was associated with an increased risk of IUGR only in individuals with BMI less than 18.5 (OR, 2.13; 95% CI, 1.22-3.73) and between 18.5 and 23.9 (OR, 2.22; 95% CI, 1.54-3.19) but not in those with BMIs of 24.0 or greater. Moreover, hypercholanemia was associated with an increased risk of IUGR in the subgroup with preeclampsia. Through interaction effects analysis, we found an additive interaction in hypercholanemia and HDP for the risk of LBW and IUGR (eTable 6 in the Supplement). There was a significant additive combined association of hypercholanemia and HDP with the risk for IUGR (Figure 2) and LBW (eFigure 3 in the Supplement). The sTBA levels in whole, early, and late pregnancy periods were associated with LBW and IUGR in both pregnant individuals with normotensive pregnancies and HDP. Furthermore, the estimated incidences of IUGR (Figure 2) and LBW (eFigure 3 in the Supplement) were steeply increased in pregnant individuals with HDP and elevated sTBA concentrations (estimated prevalence of IUGR for participants with HDP and sTBA levels of 4.1 μg/mL: 4.59%; 95% CI, 4.48%-4.70%; sTBA levels of 40.8 μg/mL: 16.75%; 95% CI, 15.39%-18.11%).

Hypercholanemia was associated with both LBW (adjusted OR, 1.29; 95% CI, 1.09-1.53) and IUGR (adjusted OR, 2.18; 95% CI, 1.62-2.91) (Table 3). Pregnant individuals with hypercholanemia and HDP had a 9.13-fold increased risk (95% CI, 6.88-12.12) of LBW and 19.14-fold increased risk (95% CI, 12.09-30.28) of IUGR compared with those with low sTBA and without HDP (Table 3). By stratification, those with high sTBA concentrations were associated with a 2.27-fold increased risk (95% CI, 1.67-3.08) of LBW and 2.21-fold increased risk (95% CI, 1.36-3.60) of IUGR in pregnancies complicated with HDP. There was also a significant combined association of maternal hypercholanemia in late pregnancy and HDP with the risk of LBW and IUGR (eTable 7 in the Supplement).

**Table 3.** Individual and Combined Association of Gestational Hypercholanemia and HDP With Risk of LBW and IUGR

Characteristic	Pregnancies, No.	LBW		IUGR	
		No. (%)	aOR (95% CI)	No. (%)	aOR (95% CI)
sTBA binary^a^					
Nonhypercholanemia	63 778	1615 (2.53)	1 [Reference]	312 (0.49)	1 [Reference]
Hypercholanemia	4467	164 (3.67)	1.29 (1.09-1.53)	62 (1.39)	2.18 (1.62-2.91)
HDP^b^					
No	66 720	1545 (2.32)	1 [Reference]	287 (0.43)	1 [Reference]
Yes	1525	234 (15.34)	4.20 (3.69-4.78)	87 (5.70)	8.74 (6.90-11.08)
Combined association^c^					
Without HDP					
Nonhypercholanemia	62 439	1432 (2.29)	1 [Reference]	247 (0.40)	1 [Reference]
Hypercholanemia	4281	113 (2.64)	1.02 (0.83-1.27)	40 (0.93)	2.18 (1.52-3.15)
With HDP					
Nonhypercholanemia	1339	183 (13.67)	3.83 (3.33-4.4)	65 (4.85)	8.84 (6.83-11.44)
Hypercholanemia	186	51 (27.42)	9.13 (6.88-12.12)	22 (11.83)	19.14 (12.09-30.28)
Stratified by HDP category^c^					
Without HDP					
Nonhypercholanemia	62 439	1432 (2.29)	1 [Reference]	247 (0.40)	1 [Reference]
Hypercholanemia	4281	113 (2.64)	1.03 (0.84-1.28)	40 (0.93)	2.18 (1.51-3.14)
With HDP					
Nonhypercholanemia	1339	183 (13.67)	1 [Reference]	65 (4.85)	1 [Reference]
Hypercholanemia	186	51 (27.42)	2.27 (1.67-3.08)	22 (11.83)	2.21 (1.36-3.60)

Abbreviations: aOR, adjusted odds ratio; HDP, hypertensive disorders in pregnancy; IUGR, intrauterine fetal growth restriction; LBW, low birth weight; sTBA serum total bile acids.

^a^ Nonhypercholamenia was defined as sTBA levels of less than 4.1 μg/mL (to convert to micromoles per liter, multiply by 2.448). Adjusted for prepregnancy body mass index, age, education level, race, parity, alanine aminotransferase level, HDP, and diabetes during pregnancy.

^b^ Adjusted for sTBA level, prepregnancy body mass index, age, education level, race, parity, alanine aminotransferase level, and diabetes during pregnancy.

^c^ Adjusted for prepregnancy body mass index, age, education level, race, parity, alanine aminotransferase level, and diabetes during pregnancy.

## Discussion

In the present study, we found that elevated sTBA levels during pregnancy could be an independent risk factor for LBW and IUGR. To our knowledge, this is the first study assessing the association between sTBA levels during pregnancy and the risk of IUGR in a large birth cohort.

Until recently, the association of hypercholanemia or ICP with fetal weight remained controversial. Previous studies in Sweden and the UK suggested that pregnancy complicated by ICP accelerates fetal growth and is associated with an increased risk of LGA.^4,18^ However, we did not find an association between hypercholanemia and the incidence of LGA in this study. In contrast, a previous study in Turkey demonstrated that the birth weights of fetuses of patients with ICP were lower than those of patients without ICP.^16^ A recent study also reported that birth weight decreased with increasing sTBA levels in a cohort of 13 801 pregnant individuals in China.^15^ In this study, we observed a J-shaped nonlinear association between sTBA and birth weight and a negative association between sTBA concentrations of 4.08 μg/mL or greater and birth weight in a larger cohort (ie, with 68 245 pregnant individuals). The inconsistencies among these studies may partially be explained by differences in sample sizes, population ethnicity, and the diagnostic criteria for ICP or gestational hypercholanemia. The proportion of pregnant individuals with tobacco and alcohol consumption in China is much lower than in European countries and the United States.^23,24^ In addition, the prevalence of individuals with overweight or obesity in the pregnant population was higher than 50% in the United States,^25^ whereas, the ratio of patients with overweight or obesity was only 12.5% in the current study. Interestingly, we found hypercholanemia was associated with an increased risk of IUGR only in those with underweight and normal weight but not in those with overweight or obesity.

A previous study reported that a reduction in fetal weight and a potential increased incidence of IUGR was observed in a gestational cholestasis animal model.^26^ However, the clinical evidence of the association between sTBA and the risk of IUGR in human pregnancy remains insufficient. In this study, we found that a high sTBA level during the whole pregnancy, especially during the late pregnancy period, was a risk factor for IUGR. As reported, ICP or gestational hypercholanemia arises mainly in the second to third trimesters of pregnancy,^1^ when a fetus undergoes rapid weight gain within intrauterine development.^27^ Thus, our findings suggest that it is meaningful to simultaneously monitor sTBA concentrations during the management of patients with potential for LBW and IUGR.

Nevertheless, the pathogenesis of elevated sTBA-induced fetal disorders remains obscure. It has been reported that the severity of adverse outcomes was closely associated with the maternal sTBA level, including preterm labor, fetal hypoxia, intracranial hemorrhage, and even sudden intrauterine death in patients with ICP.^28,29^ It is thus meaningful to find disease markers for the identification and prevention of adverse fetal outcome of patients with ICP or gestational cholestasis as early and precisely as possible. Recently, some potential parameters, including serum total ADH activity^30^ and bile acid profile,^31^ were also identified as useful for monitoring the progression of ICP. Hydrophobic bile acids can undermine the integrity of cell membranes by disrupting lipid components^32^ and promote cell apoptosis.^33,34,35^ Moreover, previous studies by us and others have shown that hydrophobic bile acid accumulation induces oxidative damage and mitochondrial dysfunction in cholestatic rodents.^32,34,36,37^ Additionally, toxic bile acids may affect placental vascular remodeling and induce vasoconstriction of placental chorionic vessels or the umbilical vein,^38,39^ thus triggering insufficient fetal perfusion, which is one of the major reasons for IUGR.^40,41^ Further studies are required to understand the underlying pathological mechanism of excessive bile acid–induced IUGR.

An unexpected observation of this study was that there was a notable combined association of HDP and hypercholanemia with the risk of IUGR, especially in the late pregnancy period. Previous studies demonstrated that HDP was among the major causes of IUGR.^40,42^ In addition, preeclampsia was associated with ICP^43,44,45^ and IUGR.^46^ Various studies have supported that dysregulated placental angiogenic factors and antiangiogenic factors contribute to the development of preeclampsia and IUGR.^47,48^ In addition, the ratio of fms-like tyrosine kinase 1 to placental growth factor in maternal serum can project both preeclampsia and IUGR.^49^ Our unpublished data also found that excessive bile acid can disrupt the expression balance of these angiogenic factors in trophoblastic cells. Thus, we proposed that ICP and HDP, especially preeclampsia, might share some common risk factors or signaling pathways that function synergistically during the pathogenesis of IUGR.

### Limitations

This study has several limitations. One limitation was that this study applied a hospital-based retrospective cohort in Shanghai with a relatively low-risk population for IUGR. A second limitation was that it remains unclear to what extent the findings can be generalized to other populations because this study included an ethnically homogeneous Chinese population with predominant Han race. Another potential limitation was that the ratio of severe cases of ICP or hypercholanemia in the current cohort was relatively low due to active follow-up and early intervention according to Chinese ICP management guidelines. In addition, because of observational nature of current study, any statements on causality of the associations were limited.

## Conclusions

The current findings suggest a significantly increased risk of LBW and IUGR was associated with a combined association of hypercholanemia and HDP. Thus, it is suggested that more attention should be paid to the recognition and management of gestational hypercholanemia, especially in pregnant individuals with HDP. Additional studies, including prospective cohort studies and randomized clinical trials, are needed to confirm the current findings and potential benefits of lowering sTBA concentrations using drugs (such as ursodesoxycholic acid) in these patients.

## Notes

Supplement. **eFigure 1.** Study Population Flowchart **eFigure 2.** Association Between Maternal sTBA Levels With Fetal Birth Weight in Individuals With sTBA of 4.1 μg/mL or Greater **eFigure 3.** Association Between Maternal sTBA Levels With Risk of LBW, Stratified by HDP **eTable 1. **Comparison of Fetal Characteristics in Pregnant Individuals With or Without Hypercholanaemia **eTable 2. **Analysis of Risk of Adverse Fetal Outcomes in Pregnant Individuals With Gestational Hypercholanaemia **eTable 3.** Analysis of LBW and IUGR Risk in Patients With Different sTBA Levels in Early and Late Pregnancy **eTable 4. **Analysis of LBW and IUGR Risk in Patients With Hypercholanaemia During Pregnancy, Stratified by BMI Categories **eTable 5. **Analysis of LBW and IUGR Risk in Patients With Gestational Hypercholanaemia, Stratified by Hypertension Status **eTable 6. **Analysis of Interaction Association of Hypercholanaemia and Preeclampsia on Risk of LBW and IUGR **eTable 7. **Individual and Combined Effect of Hypercholanaemia at Late Pregnancy and Preeclampsia on Risk of LBW and IUGR
