# Supplementary material for: Association of Elevated Maternal Serum Total Bile Acids With Low Birth Weight and Intrauterine Fetal Growth Restriction
Source: JAMA Netw Open. 2021 Jul 19;4(7):e2117409. doi: 10.1001/jamanetworkopen.2021.17409 (PMC8290304; doi:10.1001/jamanetworkopen.2021.17409)
Supplement: Supplement. — eFigure 1. Study Population Flowchart eFigure 2. Association Between Maternal sTBA Levels With Fetal Birth Weight in Individuals With sTBA of 4.1 μg/mL or Greater eFigure 3. Association Between Maternal sTBA Levels With Risk of LBW, Stratified by HDP eTable 1. Comparison of Fetal Characteristics in Pregnant Individuals With or Without Hypercholanaemia eTable 2. Analysis of Risk of Adverse Fetal Outcomes in Pregnant Individuals With Gestational Hypercholanaemia eTable 3. Analysis of LBW and IUGR Risk in Patients With Different sTBA Levels in Early and Late Pregnancy eTable 4. Analysis of LBW and IUGR Risk in Patients With Hypercholanaemia During Pregnancy, Stratified by BMI Categories eTable 5. Analysis of LBW and IUGR Risk in Patients With Gestational Hypercholanaemia, Stratified by Hypertension Status eTable 6. Analysis of Interaction Association of Hypercholanaemia and Preeclampsia on Risk of LBW and IUGR eTable 7. Individual and Combined Effect of Hypercholanaemia at Late Pregnancy and Preeclampsia on Risk of LBW and IUGR [file jamanetwopen-e2117409-s001.pdf]

Supplemental Online Content

Song F, Chen Y, Chen L, Li H, Cheng X, Wu W. Association of elevated maternal serum total bile acids with low birth weight and intrauterine fetal growth restriction. *JAMA Netw Open.* 2021;4(7):e2117409. doi:10.1001/jamanetworkopen.2021.17409

eFigure 1. Study Population Flow Chart

eFigure 2. Association Between Maternal sTBA Levels With Fetal Birth Weight in Individuals With sTBA of 4.1 µg/mL or Greater

eFigure 3. Association Between Maternal sTBA Levels With Risk of LBW, Stratified by HDP

eTable 1. Comparison of Fetal Characteristics in Pregnant Individuals With or Without Hypercholanaemia

eTable 2. Analysis of Risk of Adverse Fetal Outcomes in Pregnant Individuals With Gestational Hypercholanaemia

eTable 3. Analysis of LBW and IUGR Risk in Patients With Different sTBA Levels in Early and Late Pregnancy

eTable 4. Analysis of LBW and IUGR Risk in Patients With Hypercholanaemia During Pregnancy, Stratified by BMI Categories

eTable 5. Analysis of LBW and IUGR Risk in Patients With Gestational Hypercholanaemia, Stratified by Hypertension Status

eTable 6. Analysis of Interaction Association of Hypercholanaemia and Preeclampsia on Risk of LBW and IUGR

eTable 7. Individual and Combined Effect of Hypercholanaemia at Late Pregnancy and Preeclampsia on Risk of LBW and IUGR

This supplemental material has been provided by the authors to give readers additional information about their work.

© 2021 Song F et al. *JAMA Network Open.*

eFigure 1. Study Population Flow Chart

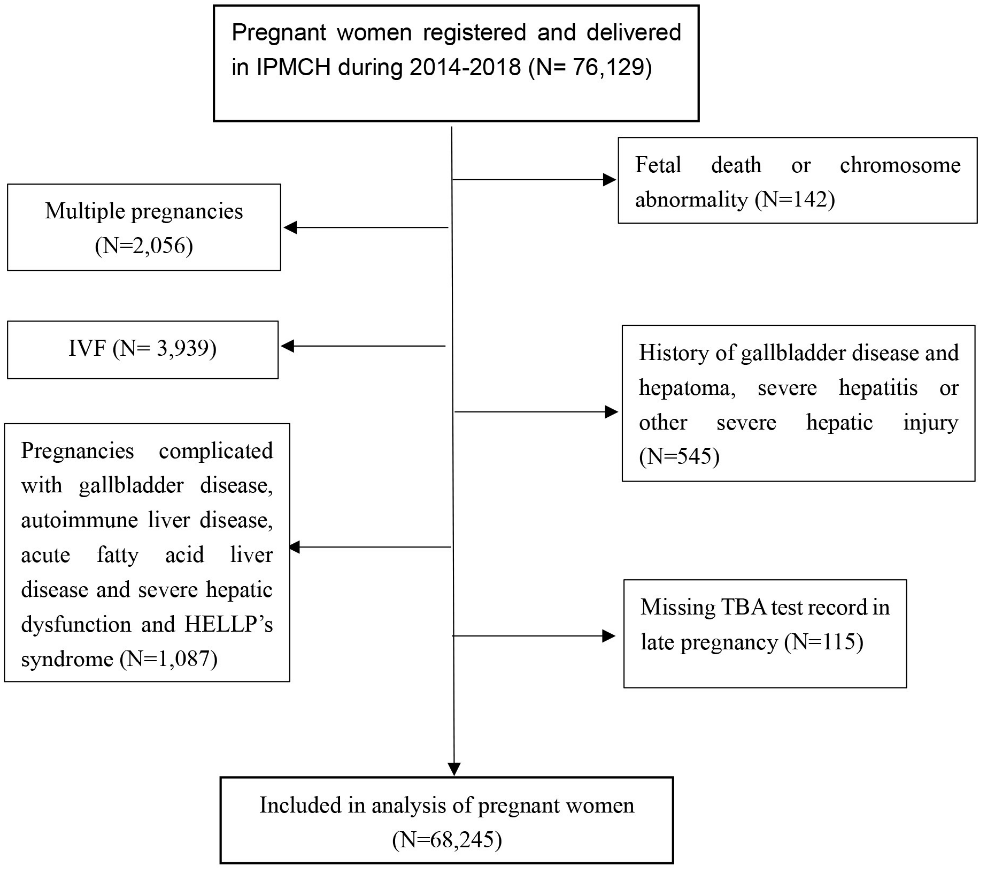

IPMCH: International Peace Maternity and Child Health Hospital; IVF: in vitro fertilization.

© 2021 Song F et al. *JAMA Network Open.*

eFigure 2. Association Between Maternal sTBA Levels With Fetal Birth Weight in Individuals With sTBA of 4.1 µg/mL or Greater

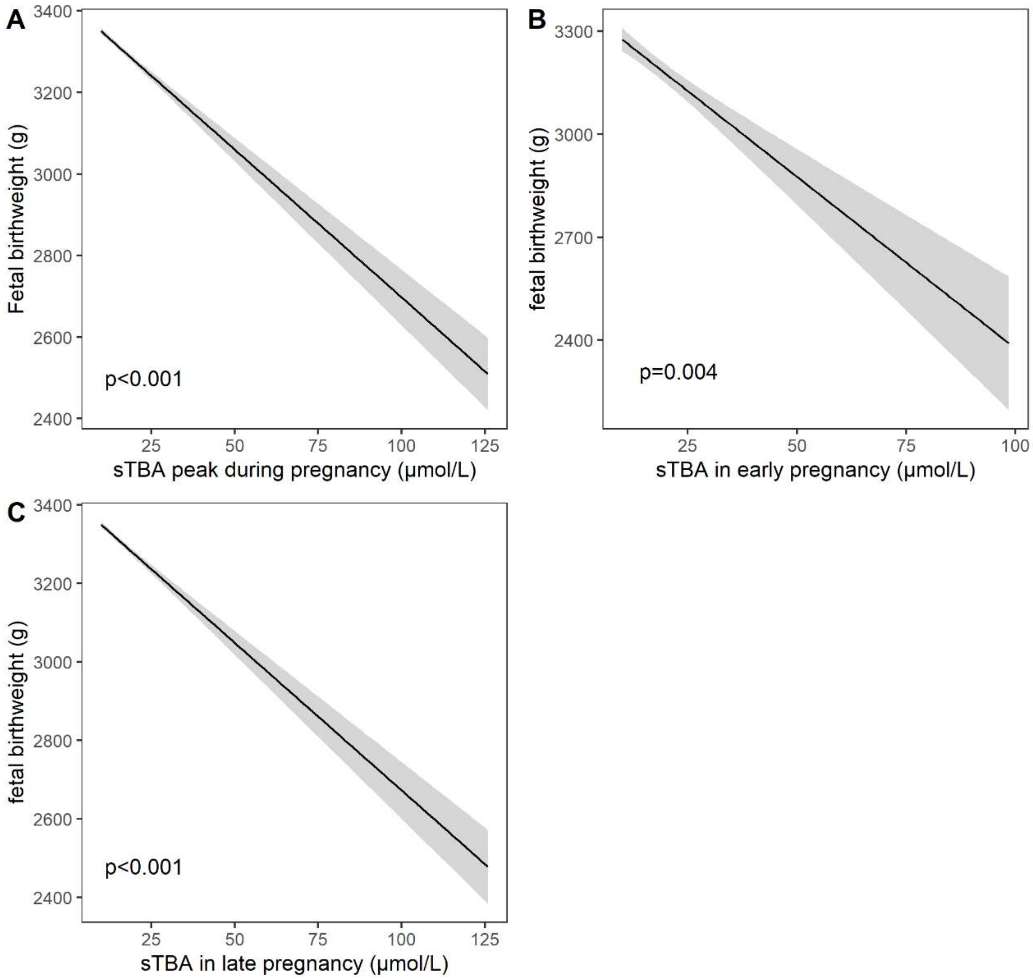

Analysis of the association between peak sTBA level during pregnancy (A), in early pregnancy (B) or in late pregnancy (C) with fetal birthweight by multiple linear regression models in subpopulation with sTBA≥10 µmol/L. Data were expressed as predicted mean birth weight with 95% CI. Adjusted for pre-pregnancy BMI, age, education levels, race, parity, HDP, diabetes mellitus during pregnancy, fetal sex, gestation age at birth, and alanine aminotransferase level.

© 2021 Song F et al. *JAMA Network Open.*

eFigure 3. Association Between Maternal sTBA Levels With Risk of LBW, Stratified by HDP

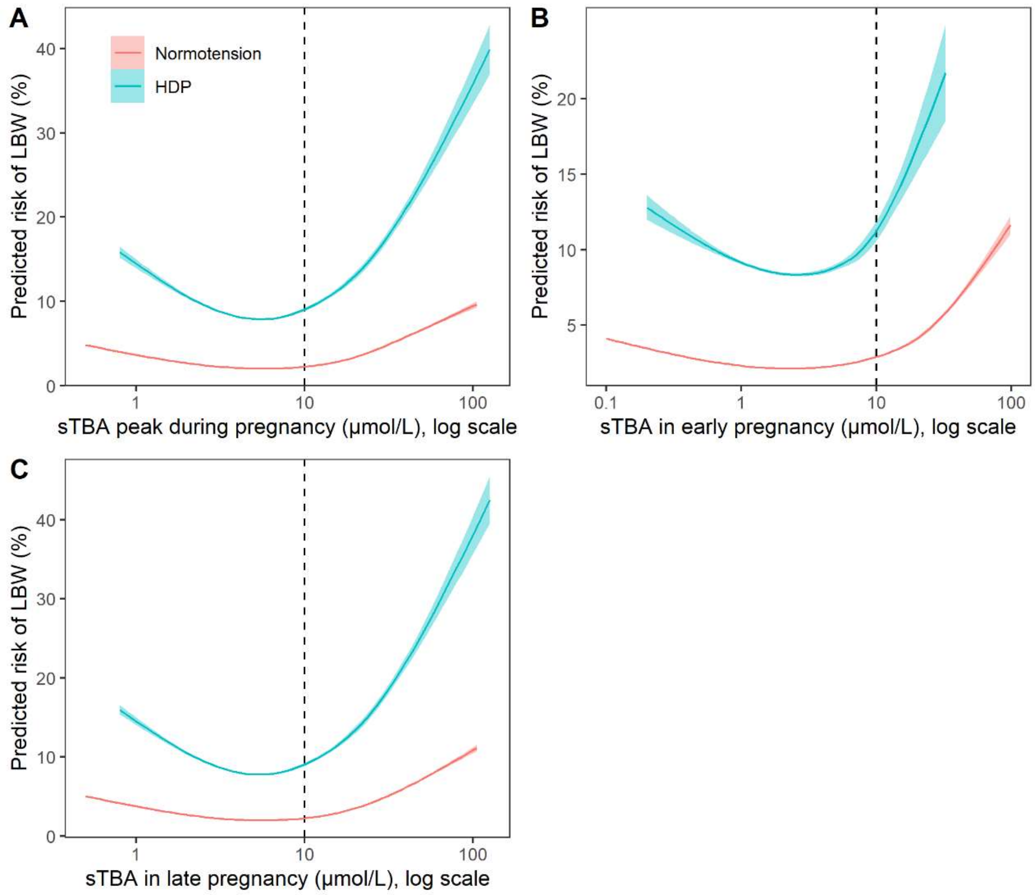

Association of risk of LBW with peak sTBA level during whole (A), in early (B) and in late (C) pregnancy periods. Data were expressed as predicted mean risk with 95% CI. Adjusted for pre-pregnancy BMI, age, education levels, race, parity, HDP, diabetes mellitus during pregnancy, and alanine aminotransferase level.

© 2021 Song F et al. *JAMA Network Open.*

eTable 1. Comparison of Fetal Characteristics in Pregnant Individuals With or Without Hypercholanaemia

Fetal characteristics	Hypercholanaemia (n=4,467)	Non-hypercholanaemia (n=63,778)	p value
Birth weight, g, mean (SD)	3,310 (463)	3,340 (427)	0.005
Body length, cm, mean (SD)	49.8 (1.53)	49.8 (1.26)	0.013
Sex (male), n (%)	2,386 (53.4%)	32,822 (51.5%)	0.012
Apgar at 1 min < 7, n (%)	40 (0.9%)	465 (0.7%)	0.24
LBW, n (%)	164 (3.7%)	1,615 (2.5%)	<0.001
Macrosomia, n (%)	252 (5.7%)	3,599 (5.6%)	1.0
SGA, n (%)	246 (5.5%)	2,532 (4.0%)	<0.001
LGA, n (%)	464 (10.4%)	6,353 (10.0%)	0.35
IUGR, n (%)	62 (1.4%)	312 (0.5%)	<0.001

LBW: low birth weight; SGA: small for gestation age; LGA: large for gestation age; IUGR: intrauterine growth restriction

© 2021 Song F et al. *JAMA Network Open.*

eTable 2. Analysis of Risk of Adverse Fetal Outcomes in Pregnant Individuals With Gestational Hypercholanaemia

**Outcomes**	**cOR (95% CI)**	**aOR [a] (95% CI)**
LBW	1.47 (1.25, 1.73)	1.29 (1.09, 1.53)
Macrosomia	1.00 (0.88, 1.14)	1.07 (0.93, 1.22)
SGA	1.41 (1.23, 1.61)	1.29 (1.12, 1.48)
LGA	1.05 (0.95, 1.16)	1.11 (0.99, 1.23)
IUGR	2.86 (2.18, 3.77)	2.18 (1.62, 2.91)

Abbreviations: LBW, low birth weight; SGA: small for gestation age; LGA: large for gestation age; IUGR, intrauterine growth restriction; cOR, crude odds ratio; aOR, adjusted odds ratio.

[a] adjusted for maternal pre-pregnancy BMI, age, education levels, race, parity, HDP, diabetes mellitus during pregnancy, alanine aminotransferase level.

© 2021 Song F et al. *JAMA Network Open.*

eTable 3. Analysis of LBW and IUGR Risk in Patients With Different sTBA Levels in Early and Late Pregnancy

		LBW		IUGR	
**sTBA, μmol/L**	**N**	**n (%)**	**aOR [a] (95% CI)**	**n (%)**	**aOR [a] (95% CI)**
Early pregnancy [b]					
< 5	64,288	1663(2.59)	1.00 (Ref)	332 (0.52)	1.00 (Ref)
5~10	2,638	67(2.54)	0.93 (0.72, 1.2)	21 (0.80)	1.52 (0.97, 2.37)
≥10	316	18(5.7)	2.25 (1.39, 3.66)	6 (1.90)	3.60 (1.56, 8.28)
P for trend		0.06		<0.001	
Late pregnancy					
< 5	40,256	1040(2.58)	1.00 (Ref)	171 (0.42)	1.00 (Ref)
5~10	23,666	579(2.45)	0.92 (0.82, 1.02)	143 (0.6)	1.29 (1.03, 1.62)
10~20	3,776	123(3.26)	1.1 (0.9, 1.34)	45 (1.19)	2.06 (1.45, 2.93)
20~40	460	28(6.09)	1.89 (1.26, 2.83)	12 (2.61)	3.84 (2.03, 7.27)
≥ 40	87	9(10.34)	3.46 (1.68, 7.09)	3 (3.45)	5.41 (1.6, 18.27)
P for trend		0.001		<0.001	

Abbreviations: LBW, low birth weight; IUGR, intrauterine growth restriction; sTBA, serum total bile acids; IUGR, intrauterine growth restriction; aOR, adjusted odds ratio.

[a] adjusted for maternal pre-pregnancy BMI, age, education levels, race, parity,

© 2021 Song F et al. *JAMA Network Open.*

alanine aminotransferase level, HDP, diabetes mellitus during pregnancy.

[b] There are 1,003 (1.47%) pregnant women with missing sTBA test in early pregnancy. Data were missing at random. No significance in ratios of LBW or IUGR between women with or without TBA missing.

© 2021 Song F et al. *JAMA Network Open.*

eTable 4. Analysis of LBW and IUGR Risk in Patients With Hypercholanaemia During Pregnancy, Stratified by BMI Categories

		LBW		IUGR	
**Characteristics**	**N**	**n (%)**	**aOR (95% CI)** [a]	**n (%)**	**aOR (95% CI)** [a]
BMI <18.5					
Non-hypercholanaemia	9,310	317 (3.4)	1.00 (Ref)	74 (0.79)	1.00 (Ref)
Hypercholanaemia	808	36 (4.46)	1.14 (0.80, 1.64)	16 (1.98)	2.13 (1.22, 3.73)
BMI 18.5~23.9					
Non-hypercholanaemia	46,340	1,063 (2.29)	1.00 (Ref)	205 (0.44)	1.00 (Ref)
Hypercholanaemia	3,280	109 (3.32)	1.31 (1.07, 1.61)	40 (1.22)	2.22 (1.54, 3.19)
BMI ≥24.0					
Non-hypercholanaemia	8,128	235 (2.89)	1.00 (Ref)	33 (0.41)	1.00 (Ref)
Hypercholanaemia	379	19 (5.01)	1.43 (0.86, 2.38)	6 (1.58)	2.12 (0.73, 6.12)

Abbreviations: LBW, low birth weight; IUGR, intrauterine growth restriction; BMI, body mass index (calculated as weight in kilograms divided by height in meters squared); aOR, adjusted odds ratio.

[a] adjusted for maternal pre-pregnancy BMI, age, education levels, race, parity, alanine aminotransferase level, hypertensive disorders in pregnancy, diabetes mellitus during pregnancy.

© 2021 Song F et al. *JAMA Network Open.*

eTable 5. Analysis of LBW and IUGR Risk in Patients With Gestational Hypercholanaemia, Stratified by Hypertension Status

		LBW		IUGR	
**Characteristics**	**N**	**n (%)**	**aOR (95% CI)** [a]	**n (%)**	**aOR (95% CI)** [a]
Normotension					
Non-hypercholanaemia	60,262	1,331 (2.21)	1.00 (Ref)	217 (0.36)	1.00 (Ref)
Hypercholanaemia	4,113	98 (2.38)	1.03 (0.84, 1.28)	37 (0.9)	2.18 (1.51, 3.14)
Preexisted hypertension					
Non-hypercholanaemia	823	57 (6.93)	1.00 (Ref)	18 (2.19)	1.00 (Ref)
Hypercholanaemia	59	5 (8.47)	1.10 (0.41, 2.94)	0 (0)	-
Gestational hypertension					
Non-hypercholanaemia	1,354	44 (3.25)	1.00 (Ref)	12 (0.89)	1.00 (Ref)
Hypercholanaemia	109	10 (9.17)	3.08 (1.48, 6.41)	3 (2.75)	2.89 (0.78, 10.79)
Preeclampsia					
Non-hypercholanaemia	1,339	183 (13.67)	1.00 (Ref)	65 (4.85)	1.00 (Ref)
Hypercholanaemia	186	51 (27.42)	2.03 (1.39, 2.96)	22 (11.83)	2.13 (1.23, 3.69)

Abbreviations: LBW, low birth weight; IUGR, intrauterine growth restriction; aOR, adjusted odds ratio.

[a] adjusted for maternal pre-pregnancy BMI, age, education levels, race, parity, alanine aminotransferase level, diabetes mellitus during pregnancy.

© 2021 Song F et al. *JAMA Network Open.*

eTable 6. Analysis of Interaction Association of Hypercholanaemia and Preeclampsia on Risk of LBW and IUGR

**Indicators of additive interaction** [a]	**LBW**	**IUGR**
RERI	5.17 (2.62, 7.72)	9 (0.43, 17.56)
AP	0.58 (0.45, 0.71)	0.48 (0.22, 0.73)
SI	2.84 (1.98, 4.09)	2.01 (1.2, 3.34)

Abbreviations: LBW, low birth weight; IUGR, intrauterine growth restriction; sTBA, serum total bile acids; RERI, relative excess risk due to interaction; AP, attributable proportion; SI, synergy index.

[a] RERI > 0, AP > 0, or SI > 1 suggest significant additive interaction.

© 2021 Song F et al. *JAMA Network Open.*

eTable 7. Individual and Combined Effect of Hypercholanaemia at Late Pregnancy and Preeclampsia on Risk of LBW and IUGR

		LBW		IUGR	
**Characteristics**	**N**	**n (%)**	**aOR (95% CI)**	**n (%)**	**aOR (95% CI)**
**sTBA binary** [a]					
Non-hypercholanaemia	63,922	1,619 (2.53)	1.00 (Ref)	314 (0.49)	1.00 (Ref
Hypercholanaemia	4,323	160 (3.7)	1.28 (1.08, 1.52)	60 (1.39)	2.10 (1.56, 2.83)
**HDP** [b]					
No	66,720	1,545 (2.32)	1.00 (Ref)	287 (0.43)	1.00 (Ref
Yes	1,525	234 (1 5.34)	4.21 (3.70, 4.79)	87 (5.70)	8.77 (6.92, 11.11)
Combined effect [c]					
**Without HDP**					
Non-hypercholanaemia	62,583	1,436 (2.29)	1.00 (Ref)	249 (0.40)	1.00 (Ref
Hypercholanaemia	4,137	109 (2.63)	1.02 (0.83, 1.27)	38 (0.92)	2.14 (1.48, 3.1)
**With HDP**					
Non-hypercholanaemia	1,339	183 (13.67)	3.82 (3.32, 4.39)	65 (4.85)	8.84 (6.83, 11.43)
Hypercholanaemia	186	51 (27.42)	8.74 (6.55, 11.65)	22 (11.83)	17.99 (11.24, 28.78)
Stratified by HDP categories [c]					
**Without HDP**					
Non-hypercholanaemia	62,583	1,436 (2.29)	1.00 (Ref)	249 (0.40)	1.00 (Ref
Hypercholanaemia	4,137	109 (2.63)	1.04 (0.84, 1.28)	38 (0.92)	2.14 (1.48, 3.11)
**With HDP**					
Non-hypercholanaemia	1,339	183 (13.67)	1.00 (Ref)	65 (4.85)	1.00 (Ref
Hypercholanaemia	186	51 (27.42)	2.18 (1.60, 2.98)	22 (11.83)	2.06 (1.25, 3.39)

Abbreviations: LBW, low birth weight; IUGR, intrauterine growth restriction;

© 2021 Song F et al. *JAMA Network Open.*

sTBA, serum total bile acids; HDP, hypertensive disorders in pregnancy; aOR, adjusted odds ratio; Non-hypercholanaemia, sTBA < 10 μmol/L; Hypercholanaemia, sTBA ≥ 10 μmol/L.

[a] adjusted for pre-pregnancy BMI, age, education levels, race, parity, alanine aminotransferase level, HDP, diabetes mellitus during pregnancy.

[b] adjusted for sTBA level, pre-pregnancy BMI, age, education levels, race, parity, alanine aminotransferase level, diabetes mellitus during pregnancy.

[c] adjusted for pre-pregnancy BMI, age, education levels, race, parity, alanine aminotransferase level, diabetes mellitus during pregnancy.

© 2021 Song F et al. *JAMA Network Open.*
